# Differences in the use of medicines for peptic ulcer and gastro-esophageal reflux disease between Serbia, Croatia and Sweden

**DOI:** 10.1186/2050-6511-13-S1-A4

**Published:** 2012-09-17

**Authors:** Bojan Stanimirov, Karmen Stankov, Nebojša Pavlović, Milica Paut Kusturica, Maja Stojančević, Ana Sabo, Momir Mikov

**Affiliations:** 1 Department of Pharmacology, Toxicology and Clinical Pharmacology, Faculty of Medicine, University of Novi Sad, 21000 Novi Sad, Serbia; 2 Clinical Centre of Vojvodina, Faculty of Medicine, University of Novi Sad, 21000 Novi Sad, Serbia

## Background

The medicines for peptic ulcer and gastro-esophageal reflux disease (ATC subgroup A02B) are among the most commonly prescribed class of drugs. The aim of this study was to analyze the pattern of consumption of histamine H_2_ receptor antagonists (H_2_RAs) and proton pump inhibitors (PPIs) in Serbia in 2010 in comparison with Croatia and Sweden.

## Methods

The data on the consumption of medicines have been provided from the databases of the national regulatory agencies. The results were expressed as the number of defined daily doses per 1000 inhabitants per day (DID). A qualitative analysis was carried out according to the drug utilization 90% (DU90%) approach.

## Results

The overall consumption of medicines from A02B subgroup in 2010 in Serbia was 22.9 DID, whereas in Croatia and Sweden was 32.8 DID and 48.6 DID, respectively. In Serbia, H_2_RAs accounted for 71.8% (16.5 DID) of medicines used within A02B subgroup, while in Croatia H_2_RAs accounted for 37.3% (12.2 DID) and in Sweden 2.2% (1.1 DID). In the same year, the utilization of PPIs in Serbia (6.5 DID) was more than three times lower than in Croatia (20.6 DID) and more than seven times lower than in Sweden (47.3 DID). The bulk of prescription (DU90%) was made up of 3 (out of 7) medicines in Serbia, 5 (out of 8) medicines in Croatia and 5 (out of 14) medicines in Sweden. The most frequently used medicine from the A02B subgroup in Serbia was ranitidine (56.0%, i.e. 12.8 DID), in Croatia pantoprazole (36.5%, i.e.12.0 DID) and in Sweden omeprazole (81.3%, i.e. 39.0 DID).

## Conclusions

The overall utilization of the medicines for peptic ulcer and gastro-esophageal reflux disease was notably lower in Serbia in comparison with Croatia and Sweden. Besides the quantity, the pattern of use showed remarkable differences. Most commonly used medicines from the A02B subgroup in Serbia were H_2_RAs whereas in Croatia and Sweden were PPIs. These findings suggest that implementation of pharmacotherapeutic guidelines in Serbia is needed in order to achieve harmonization in prescribing practice.

